# Vitamin D Status and Analysis of Specific Correlates in Preschool Children: A Cross-Sectional Study in Southern Croatia

**DOI:** 10.3390/ijerph15112503

**Published:** 2018-11-08

**Authors:** Zeljka Karin, Barbara Gilic, Daniela Supe Domic, Zdenko Sarac, Katarina Ercegovic, Natasa Zenic, Ognjen Uljevic, Mia Peric, Josko Markic

**Affiliations:** 1 Teaching Institute of Public Health of Split Dalmatian County, 21000 Split, Croatia; karinzeljka@gmail.com (Z.K.); kercegovic75@gmail.com (K.E.); 2 Faculty of Kinesiology, University of Split, 21000 Split, Croatia; barbara.gilic@outlook.com (B.G.); ouljevic@kifst.hr (O.U.); mia.peric@kifst.hr (M.P.); 3 Department of Medical Laboratory Diagnostics, University Hospital of Split, 21000 Split, Croatia; daniela.supedomic@gmail.com; 4 School of Medicine, University of Mostar, 88000 Mostar, Bosnia and Herzegovina; z-sarac@hotmail.com; 5 Department of Pediatrics, University Hospital of Split, 21000 Split, Croatia; jmarkic@mefst.hr; 6 School of Medicine, University of Split, 21000 Split, Croatia

**Keywords:** 25(OH)D, prevalence, physical activity, preschool children, body mass index

## Abstract

Vitamin D deficiency is a globally important problem, particularly in children, but there is a lack of information regarding this deficiency in preschool children from southeastern Europe. This study aimed to establish the levels of 25-hydroxyvitamin D (25(OH)D) and associations of gender, time spent outdoors, physical activity (PA), and body mass index (as predictors) with the 25(OH)D level (outcome) in healthy preschool children. The participants were preschoolers (all 5–6 years of age) from southern Croatia. All the participants were tested during their mandatory medical examination 6–7 months prior to school enrollment. The PA was obtained using the preschool-age physical activity questionnaire (Pre-PAQ), which categorizes PA into five levels (from sedentary to vigorous PA). The prevalence of 25(OH)D deficiency was high: 58% of the children had 25(OH)D levels of <50 nmol/L (deficiency), and an additional 29% had an insufficient level of 25(OH)D (50–75 nmol/L). Boys had higher levels of 25(OH)D than girls. A multinomial regression using 25(OH)D categories as the outcome and a sufficient level (>75 nmol/L) as the reference value identified gender as the only significant predictor of 25(OH)D status, with boys being at lower risk for 25(OH)D deficiency than girls. These results showed a high prevalence of 25(OH)D deficiency in preschoolers from the southern part of Croatia, which is additionally alarming based on the geographical position of the studied region (42° N) and its high number of sunshine hours (>2600 h per year). Future studies examining other potential correlates of 25(OH)D in the region are warranted.

## 1. Introduction

Vitamin D is mostly known as a crucial factor in bone health but is also related to hypertension, metabolic syndrome, autoimmune and infectious diseases, and even cancer [1,2,3]. The vitamin D status is based on the serum level of 25-hydroxyvitamin D (25(OH)D), and studies have provided evidence showing that 25(OH)D deficiency differs across regions of the world [4,5,6,7]. Deficiency of 25(OH)D is particularly important in children [8]. More precisely, although it was originally hypothesized that children were not at risk of vitamin D deficiency due to their dietary intake and outdoor activities, more recent studies have identified a high prevalence of 25(OH)D insufficiency in children and adolescents worldwide [6,9,10,11]. Moreover, studies have frequently demonstrated an inverse association between obesity indicators (i.e., body mass index and body fat measures) and 25(OH)D status, with a higher prevalence of 25(OH)D deficiency among obese/overweight children [8,12,13]. In general, it is clear that failure to address a child’s low 25(OH)D status could have serious long-term negative health implications [14]. As a result, an evident increase in the body of knowledge on factors that might be associated with 25(OH)D status in children has been observed in recent years [15,16,17].

Physical activity (PA) is any movement of the body produced by skeletal muscles that results in a greater energy expenditure than that observed at rest levels [18]. The benefits of increased levels of PA are numerous and well documented and include various physiological benefits (i.e., improved cardiovascular health status, lowered blood pressure, and better hormonal function) and psychological improvements (i.e., reduced mental stress and anxiety, improved self-esteem, and socialization) [19,20]. Additionally, the level of PA is reportedly positively correlated with 25(OH)D status. In a study in which the PA level was self-reported, the researchers obtained a positive correlation between PA and 25(OH)D status in U.S. adults (>20 years) [21], and similar conclusions were reached in other studies [22,23,24]. Although outdoor PA is particularly beneficial, even indoor PA has been recognized as beneficial with regard to 25(OH)D status [21,23].

In children, PA is even more important because it positively influences cognitive and emotional functions [25], and appropriate PA in childhood has the potential to reduce the risk of obesity [26]. Recent years have seen an increase in public interest in the relationship between PA and 25(OH)D status in children, mostly because studies completed over the last decade have regularly confirmed a high prevalence of 25(OH)D deficiency in children and adolescents [9,27]. However, the results of studies that specifically investigated a possible correlation between PA and 25(OH)D status in children are not consistent [16,17,28,29].

In brief, in a German study, PA was not correlated with 25(OH)D status in 11- to 13-year-old children [29], which is consistent with the results of a Danish study that observed eight- to 11-year-old children [28]. In contrast, another Danish study reported a positive association between PA and 25(OH)D status in younger children (aged 4–8 years) [16], and similar correlations and potentially positive effects of increased PA on 25(OH)D status were observed in children and adolescents from other world regions [9,17,30,31]. Finally, in a very recent study, Brazilian researchers reported a gender-specific association between PA and 25(OH)D status in 12- to 17-year-old adolescents; specifically, these researchers showed that PA is significantly correlated with 25(OH)D status only in boys [15]. Collectively, it appears that the association between PA and 25(OH)D status in children is not universal and should be identified specifically by (i) gender (i.e., some studies reported evidence of gender-specific associations) [15], (ii) narrow age groups (due to the specific influence of different developmental factors on 25(OH)D status in different age groups) [16,28], and (iii) geographical regions/countries (mainly due to differences in sunshine hours among different geographical locations) [9,18,27].

Croatia is located in the southeastern part of Europe, and during the last decade, the 25(OH)D status has received increasing attention in studies that investigated various populations in Croatia [32,33]. However, these studies mostly examined adolescent or adult populations or very specific samples of children (i.e., children at risk for type 1 diabetes) [34,35], and the 25(OH)D status and its correlates in apparently healthy Croatian children have not been investigated. Indeed, even in the very recent official guidelines, national public health authorities noted a lack of empirical evidence that could provide specific insights into the prevalence and correlates of 25(OH)D status in Croatia [36]. Moreover, few studies have investigated this problem in the region where Croatia is located (i.e., territory of the former Yugoslavia) [37,38,39]. Finally, to the best of our knowledge, no investigation on this territory has examined either the 25(OH)D status specifically in preschool children or the possible associations between PA and 25(OH)D status in this highly vulnerable population.

Thus, this study aimed to investigate the prevalence of 25(OH)D deficiency in healthy preschool children from the southern part of Croatia and the gender-specific associations of time spent outdoors, PA levels, and body mass index (BMI; an indicator of obesity) with 25(OH)D level in this population. Initially, we hypothesized that the PA levels and time spent outdoors would be positively correlated with 25(OH)D status, whereas the BMI would be negatively correlated with 25(OH)D status. We also hypothesized that these associations would be irrespective of gender. Due to the lack of studies on these problems in Croatia and surrounding countries, the results should help raise public awareness of the problem and contribute to a better understanding of the possible influence of the studied correlates on 25(OH)D status in this age group.

## 2. Materials and Methods

### 2.1. Participants

The participants in this cross-sectional study were healthy preschool-age children from the southern part of Croatia (age range: 5–6 years; mean age: 6.0 ± 0.4 years; *n* = 260; 128 females). All the children were tested during their regular and mandatory medical examination prior to school enrollment. Prior to the study, the investigation and protocol were approved by the Ethical Board of University of Split, School of Medicine (EBO: 2181-198-03-04-16-0009), and parent(s) were informed of the study purposes and of the potential risks and benefits of participation. The testing was performed during March and April 2017 after written parental consent was obtained. Although this study included an analysis of PA (see Section 2.2 for details), which is not a regular part of the medical examination, the response rate was 84%. In this study, we included only those children who were identified as healthy with regard to their participation in the regular scholastic system in Croatia (participation in the first year of elementary school starting the following September, approximately six months after testing). Based on (i) an estimated 70% prevalence of 25(OH)D deficiency/insufficiency [37], (ii) a theoretical population sample of approximately 3000 preschool children in the studied region, and (iii) a significance level of *p* < 0.05, the sample size required for this investigation was calculated to be 250 participants.

### 2.2. Variables

The variables investigated in this study included the child’s gender (male or female, as indicated by the accompanying parent), body mass index, indicators of PA status, and time spent outdoors, which were treated as independent variables, as well as the 25(OH)D status, which was treated as the dependent variable.

After a 6–8 h fasting period, a 5 mL venous blood sample was collected in a BD Vacutainer^®^ SSTII Advance (BD, Plymouth, UK) container. The sample was centrifuged and stored at −20 °C until its analysis at the accredited laboratory of the University Hospital of Split (Split, Croatia). The 25(OH)D levels were measured using a commercially available Elecsys^®^ Vitamin D total assay with a Cobas e601 analyzer (Roche Diagnostics International Ltd., Rotkreuz, Switzerland), which uses a competitive electrochemiluminescence binding technique. This assay employs a vitamin D-binding protein as the capture protein to bind vitamin D3(25-OH) and vitamin D2(25-OH). The detection range of the test is 7.5–175 nmol/L 25(OH)D, and the sensitivity of the assay is 5 nmol 25(OH)D/L. The intraclass CV was 5.3% at 39 nmol 25(OH)D/L, 5.6% at 67.1 nmol 25(OH)D/L, 6.7% at 165 nmol 25(OH)D/L, 2.2% at 174 nmol 25(OH)D/L, 3.9% at 70.8 nmol 25(OH)D/L, and 5.2% at 39.5 nmol 25(OH)D/L. In addition, the Limit of Blank, Limit of Detection, and Limit of Quantification were 5.0 nmol/L, 7.5 nmol/L and 12.5 nmol/L, respectively. All the samples were analyzed in duplicate. The method was standardized based on international standards [40,41].

The 25(OH)D measurement results are presented as raw values (nmol/L). Moreover, for the interpretation and identification of 25(OH)D levels, the raw data were further categorized into three categories (e.g., vitamin D sufficiency, insufficiency and deficiency). Specifically, although there is no absolute consensus regarding 25(OH)D status, we used the regularly used values of ≥75 nmol/L, 51–75 nmol/L, and <50 nmol/L for the classification of vitamin D sufficiency, insufficiency and deficiency, respectively [42]. Such categorization was also used for some of the statistical analyses (see details in Section 2.3). Additionally, a value of <25 mmol/L was considered to indicate severe vitamin D deficiency [43].

The BMI was calculated based on the measured body height (BH) and body mass (BM) using Equation (1) [44].

(1) $$\mathrm{BMI}=\frac{\mathrm{BM}\;\left(\mathrm{kg}\right)}{\mathrm{BH}\;{\left(m\right)}^{2}}$$

The BH and BM were measured using a standardized stadiometer and scale, respectively, and these measurements were performed by experienced technicians. To avoid diurnal variation, all the children were tested at the same time of the day (between 7 and 9 a.m.). The BH was measured to the nearest 1 cm, and BM was reported to the nearest 0.1 kg.

The time spent outdoors was reported by the parents as the number of hours their child spent outdoors during the last week (including the weekend).

The indicators of PA included variables obtained from the preschool-age physical activity questionnaire (Pre-PAQ), which is a reliable and valid measuring tool that aim to evaluate different activity levels of preschool children [45,46,47]. In general, the Pre-PAQ categorizes activity into five progressive levels (L1: stationary with no movement, L2: stationary with limb or trunk movement, L3: slow activity, L4: medium activity, and L5: fast-paced activity). In addition to analyzing all five levels, L1 and L2 (stationary activities) were also combined (L1 + L2) in this study, as previously suggested [45]. The Pre-PAQ (in the Croatian language) was completed by the parent who accompanied the child to the medical examination. The questionnaire was translated to the Croatian language by a native speaker of both English and Croatian (the Pre-PAQ was originally developed in English by Australian authors), and the translation was then checked by three academicians who participated in this study (two medical doctors and one exercise scientist). Although the reliability of the Pre-PAQ was previously proven to be appropriate (as detailed in previous studies [45,48]), for the purpose of this study, the test-retest reliability of the Pre-PAQ was checked by asking 25 parents to complete the questionnaire twice within a time frame of 15 days. The calculated Pearson’s correlation coefficients between the results ranged from 0.73 (for L4) to 0.81 (for L5), indicating that the appropriate to high reliability of the questionnaire.

### 2.3. Statistics

First, we calculated descriptive statistics for all the variables. Specifically, for parametric variables, we calculated the means and standard deviations, whereas frequencies (counts) and percentages are reported for nonparametric variables. The differences between boys and girls were then analyzed using *t*-test for independent samples (for parametric variables) and χ^2^ test (for categorical variables). The associations among the variables were established through Pearson’s product moment correlation coefficients, and the associations between the independent variables and 25(OH)D levels (outcome) were analyzed by multinomial logistic regression. For this statistical analysis, the outcome comprised three results/categories, namely, vitamin D sufficiency, vitamin D insufficiency, and vitamin D deficiency, and vitamin D sufficiency (>75 nmol/L) was used as the reference value. Based on the findings of previous studies that identified gender as a significant covariate of 25(OH)D [15], the analyses were performed for the total sample and then stratified for gender. Statistica for Windows version 13 (Dell Inc., Tulsa, OK, USA) was used for all the calculations, and a *p* value of <0.05 was considered significant.

## 3. Results

The descriptive statistics for the measured variables for the total population and the two genders are presented in Table 1. Boys had significantly higher levels of vitamin D (*t*-value: 2.52, *p* < 0.01) and higher numbers of PA scores of L2 (*t*-value: 2.34: *p* < 0.05) and L5 (*t*-value: 2.25, *p* < 0.05) compared with girls.

**Figure 1:**
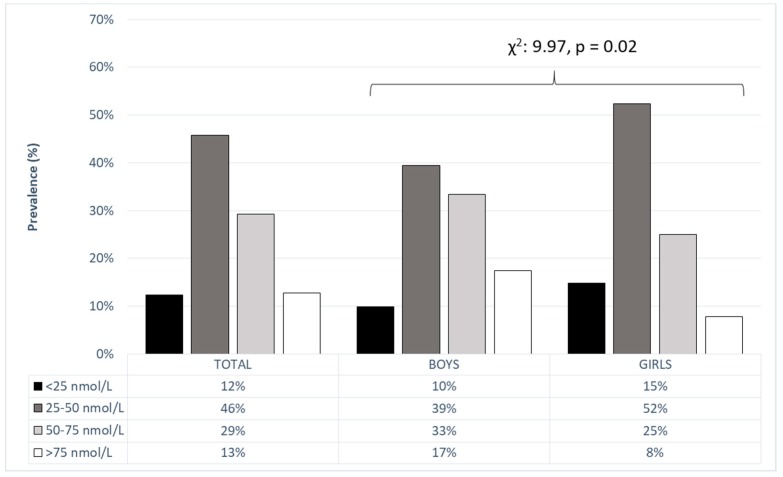
25(OH)D levels in preschool children with differences between genders (Chi square (χ^2^) and level of significance (*p*)).

In the total sample (e.g., not stratified by gender), severe 25(OH)D deficiency was evident in 12% of the participants, and an additional 46% of the participants presented with 25(OH)D levels of 25–50 mmol/L (Figure 1). The prevalence of vitamin D deficiency was higher in girls: 17% of the boys and 8% of the girls had sufficient levels of 25(OH)D (χ^2^: 9.97, *p* < 0.02).

The Pearson’s product moment correlation coefficients between the independent variables and 25(OH)D levels (outcome) did not reach statistical significance in either the total sample or the gender-based subsamples (Table 2).

The multinomial regression analysis performed using 25(OH)D status as the outcome (Table 3) confirmed the previously reported χ^2^ results. In brief, in the total sample of participants, gender was the only significant predictor of the 25(OH)D status, and boys were at lower risk for vitamin D deficiency (odds ratio (OR): 0.33; 95% confidence interval (CI): 0.15–0.74) than girls. Moreover, 25(OH)D level was not influenced by the PA levels, BMI, or time spent outdoors, and this finding was obtained for the total population of participants and for boys (Table 4) and girls (Table 5). 

## 4. Discussion

Our results showed a high prevalence of 25(OH)D deficiency in the studied population of preschool children. Additionally, boys were more physically active and had higher levels of 25(OH)D than girls. Moreover, the PA levels, BMI, and time spent outdoors (predictors) were not significantly correlated with the 25(OH)D level in the studied sample of preschool children. Therefore, our initial study hypotheses on the significant associations between predictors and 25(OH)D were rejected.

### 4.1. 25(OH)D Level in Croatian Preschool Children

The reported results showed alarming figures for the 25(OH)D status in children from the southern part of Croatia. 25(OH)D deficiency was evident among 58% of the participants, and a higher prevalence of 25(OH)D deficiency was observed in girls (49% and 67% for boys and girls, respectively). Although the high prevalence of 25(OH)D deficiency is disturbing, these results are in accordance with those of previous studies performed in similar samples of participants around the globe. Specifically, a very recent Iranian study reported subnormal 25(OH)D levels in 69% of children aged 30–72 months [11], which is similar to the prevalence reported by U.S. authors for a representative sample of one- to 11-year-old children in the period 2001–2016 [43]. However, a Danish study that investigated four- to eight-year-old children reported a lower prevalence of 25(OH)D deficiency (e.g., approximately 30% children with a 25(OH)D level of <50 nmol/L) [16]. This difference between the results presented here and the results obtained in the Danish study are particularly intriguing due to the latitude difference between Croatia and Denmark (the latitudes of these countries are approximately 42° N and 55° N, respectively). Due to its northern latitude and thus fewer hours of sunshine, it would be expected that Denmark would have a higher prevalence of 25(OH)D deficiency. However, it appears that other factors (i.e., time spent outdoors, sun-protective behavior, and dietary intake) are more significant influencing factors on 25(OH)D level than latitude and the consequent number of sunshine hours [49].

To the best of our knowledge, this study constitutes the first investigation of the 25(OH)D levels specifically in preschool children in Croatia and the surrounding countries (the territory of the former Yugoslavia). Additionally, studies performed on other samples in Croatia (i.e., adults and adolescents) mostly focused on specific populations with severe health conditions, which makes comparison of the results additionally difficult [50,51]. Therefore, we could not specifically compare our results with those previously reported for children, and future studies are needed to confirm our results. However, it appears that 25(OH)D deficiency should be recognized as a problem in this territory. Specifically, although we could not find representative data for Croatia, our colleagues from Bosnia and Herzegovina recently reported alarming figures for 25(OH)D deficiency in a representative sample from their country [37]. In short, although these studies observed a sufficient 25(OH)D level in only 20% of the tested participants, only 34% of the participants younger than 18 years had a sufficient serum level of 25(OH)D [37]. It must be stated that this cited study included participants across all age categories, and therefore, the results are not absolutely comparable to ours because we exclusively investigated preschool children. However, because Croatia, Bosnia and Herzegovina are located within the same territory and share a similar cultural and social background, which results in similar life habits (i.e., time spent outdoors and sun-protection, nutrition), the results are highly indicative and note the necessity of a more profound investigation of the problem of 25(OH)D deficiency in the whole territory.

### 4.2. Gender Differences in the 25(OH)D and Physical Activity Levels

Our study found that boys had higher levels of 25(OH)D than girls. In general, gender differences in the 25(OH)D status of children are not frequently reported, and most previous studies have shown no significant differences in 25(OH)D levels between boys and girls. For example, investigations of Asian children until early adolescence showed no significant differences in 25(OH)D levels between boys and girls [52,53,54,55], which is consistent with the results of Icelandic and Finnish studies of six- to eight-year-old children [10,56]. Moreover, the previously cited Danish study reported female gender as one of the risk factors for 25(OH)D deficiency in eight- to 11-year-old children [28]. However, it must be stated that in the previously cited studies in which the authors did not confirm gender differences, the male participants exhibited somewhat higher levels of 25(OH)D than the female participants, but these differences did not reach statistical significance [10,52,53,54,55,56].

The differences in PA levels between genders might help explain the relative inconsistency between our results and those of previous studies in which the authors reported no influence of gender on 25(OH)D status. In short, in our study, boys had higher PA levels of L2 and L5 than girls, which could also influence the 25(OH)D levels in the total population of children. Although the present study is the first to use the Pre-PAQ for measuring the PA of children and one of the first to report the PA of preschool children in the region, our results are in accordance with those of previous studies that investigated similarly aged children in other countries, although in some of them, the gender differences appeared to be significant at a somewhat older age (e.g., 10–12 years) [57,58]. Although the interpretation of gender differences in PA is beyond the scope of this paper, it is possible that such differences influence even the differences in 25(OH)D levels between genders discussed previously.

### 4.3. Lack of Association of Body Mass Index, Physical Activity and Time Spent Outdoors with 25(OH)D Levels

Although sometimes criticized as not being an objective measure of body composition (i.e., both muscle mass and adiposity can increase the BMI numerical value), the BMI is still widely used as an indicator of being overweight and an indirect indicator adiposity, particularly during childhood [59]. The association between adiposity (e.g., high BMI values) and 25(OH)D levels has been frequently confirmed. Specifically, studies have indicated an inverse association between 25(OH)D concentrations and indicators of obesity in children [60,61,62], and this association is generally explained by metabolic pathways (i.e., vitamin D affects lipolysis and adipogenesis in human adipocytes through its role in regulating the intracellular calcium concentrations) [63]. However, in our study, the 25(OH)D level was not correlated with BMI in either preschool boys or preschool girls.

To explain this inconsistency between our findings (e.g., no significant association between BMI and 25(OH)D) and the results of previous studies that found negative correlations between adiposity and 25(OH)D levels, two specific issues should be highlighted. First, our study sample of preschool children had a relatively low proportion of overweight and obese children (i.e., only 1% of the participants had values of >25 kg/m^2^, which is used as the threshold to indicate being overweight/obese), and this relatively low BMI variability likely decreased the influence of the BMI on 25(OH)D levels. In contrast, previous studies examined samples with higher prevalence of overweight/obesity, and consequently, the association between indicators of adiposity and 25(OH)D levels was more easily observed [60,61,62,64]. Second, prior studies rarely examined preschool children, and in most investigations where preschoolers were included, they formed part of the larger sample (i.e., preschool and school-age children) [64]. In addition, in previous investigations of only preschoolers, indicators of obesity were not related to 25(OH)D levels [65]. Therefore, although the relationship between indicators of adiposity and 25(OH)D status has been frequently confirmed in somewhat older children and adolescents, whether it truly exists in this age group remains questionable [60,61,62,64].

Although some previous studies reported potential beneficial effects of physical activity on 25(OH)D levels in children and adolescents [9,15], our results did not confirm such an association among preschoolers from southern Croatia. To explain our findings, several issues deserve attention. The first explanation for the lack of an association between PA and 25(OH)D levels is specific to the participant sample. Briefly, this study enrolled children in a very narrow age range (5–6 years), and it is possible that some other factors are more important determinants of 25(OH)D levels in such participant samples. Second, the PA level was determined using the Pre-PAQ. As explained in the Methods section, the parents answered the questionnaire for their children, and their participation was voluntary. It is thus possible that the parents did not objectively report their child’s PA either because (i) they were personally unaware of their child’s PA-related behavior or (ii) due to a tendency to provide ocially desirable responses (i.e., because physical activity is “socially desirable behavior”, parents will report a higher level of vigorous physical activity for their child) [66].

The “social desirability bias” is also a probable explanation for the lack of an association between sun-exposure time and 25(OH)D levels. Specifically, until recently, the problem of 25(OH)D deficiency was not particularly discussed in the Croatian scientific and professional literature because of Croatia’s geographical location (42° N) and its high number of sunshine hours (>2600 h per year). However, recent epidemiological data have provided evidence indicating low 25(OH)D levels even in southern countries [67]. Consequently, the importance of sun-exposure time in the prevention of 25(OH)D deficiency has become a widely known issue in our country [36], and an increased level of sun exposure has thus become a certain type of “socially desirable behavior”, similar to physical activity. This trend is particularly apparent in parents with regard to their children’s sun-exposure time. Consequently, we may logically question the objectivity of the parents’ reporting of their children’s sun-exposure time. Additionally, we cannot ignore the fact that time spent outdoors was not reported at the same moment for all participants (i.e., children were tested during March), which might result in differences due to weather conditions between the weeks when children were tested.

### 4.4. Limitations and Strengths

In addition to the study limitations discussed previously (i.e., social-desirability bias and parental unawareness of the children’s behavior), this study has some additional limitations. First, in this study, we used the children’s BMI as the indicator of anthropometric status, and future studies should observe the association between body composition and 25(OH)D levels using more precise measures of adiposity and body build. Next, knowing the possible influence of dietary intake on 25(OH)D levels, the lack of data on dietary intake is a significant limitation of the study. Additionally, this study observed children from only one region (the Mediterranean part of Croatia). Because of the differences in other factors that might influence 25(OH)D levels in preschool children (i.e., type of diet and 25(OH)D supplementation), the generalization of these results is relatively limited.

This study is one of the first to investigate 25(OH)D levels and some of its correlates specifically in healthy preschool children from the region and is likely the first to address this issue in Croatia. The 25(OH)D levels of all participants were determined within a relatively short time span, and the samples were analyzed in only one (accredited) laboratory. The sample of participants included children in a very narrow age range (5–6 years), and the results may be considered plausible for the studied sample. Finally, the gender-specific approach is one of the important strengths of this investigation. Therefore, despite the previously mentioned limitations, the results and observations presented could be used in future, more profound investigations of this problem.

## 5. Conclusions

Our results showed that 25(OH)D deficiency is very prevalent in apparently healthy preschool children aged 5–6 years from the southern part of Croatia. Specifically, 25(OH)D deficiency was evident in almost 60% of children (49% of males and 67% of females). Gender was a strong factor influencing 25(OH)D levels, with boys at lower risk for 25(OH)D deficiency than girls. Although the results showed that girls were also less physically active than boys, we could not confirm that PA level is a factor that significantly influences the 25(OH)D levels in the observed children. The latter conclusion could be additionally supported by the fact that the BMI, as an indicator of adiposity, was also not related to the 25(OH)D level. Finally, we did not find an association between time spent outdoors and the 25(OH)D level.

Because this study observed children from one specific geographical region (i.e., the Mediterranean part of Croatia), which is characterized by a mild climate and a high number of sunshine hours, the results should be confirmed in other regions. Additionally, in further studies, special attention should be paid to other potential correlates of 25(OH)D levels, such as sun-protective behavior (i.e., usage of sun-protective creams, lotions, and clothing), type of diet, and 25(OH)D supplementation.

## Figures and Tables

**Table 1:** Descriptive statistics for the observed variables with differences between genders (*t*-test for independent samples).

	Total	Boys	Girls	*t*-Test
	(*n* = 260)	(*n* = 132)	(*n* = 128)	
	Mean ± SD	Mean ± SD	Mean ± SD	*t*-Value (*p*)
25(OH)D (nmol/L)	46.55 ± 20.16	49.62 ± 19.68	43.38 ± 20.24	2.52 (0.01)
BMI (kg/m^2^)	15.91 ± 2.16	15.91 ± 2.14	15.92 ± 2.19	−0.04 (0.97)
Pre-PAQ/L1 (score)	1.15 ± 0.83	1.11 ± 0.79	1.18 ± 0.88	−0.66 (0.51)
Pre-PAQ/L2 (score)	0.80 ± 0.68	0.90 ± 0.69	0.71 ± 0.65	2.34 (0.02)
Pre-PAQ/L1 + L2 (score)	1.95 ± 1.16	2.02 ± 1.15	1.89 ± 1.17	0.87 (0.38)
Pre-PAQ/L3 (score)	0.96 ± 0.67	0.94 ± 0.69	0.98 ± 0.65	−0.52 (0.60)
Pre-PAQ/L4 (score)	0.92 ± 0.73	0.94 ± 0.77	0.89 ± 0.69	0.65 (0.52)
Pre-PAQ/L5 (score)	0.32 ± 0.45	0.39 ± 0.49	0.26 ± 0.4	2.25 (0.03)
Time spent outdoors (hours/week)	17.42 ± 7.12	17.94 ± 7.61	16.88 ± 6.57	1.21 (0.23)

Pre-PAQ—preschool-age physical activity questionnaire; Pre-PAQ/L1-L5—levels of physical activity as indicated by the Pre-PAQ; BMI—body mass index; SD—standard deviation.

**Table 2:** Univariate associations between independent variables and 25(OH)D level.

Independent Variables	25(OH)D (nmol/L)					
	Total		Boys		Girls	
	(*n* = 260)		(*n* = 132)		(*n* = 128)	
	*r*	*p*	*r*	*p*	*r*	*p*
BMI (kg/m^2^)	−0.04	0.49	0.01	0.85	−0.01	0.25
Pre-PAQ/L1 (score)	−0.03	0.59	−0.08	0.38	0.02	0.86
Pre-PAQ/L2 (score)	−0.07	0.25	−0.07	0.42	−0.12	0.17
Pre-PAQ/L1 + L2 (score)	−0.06	0.29	−0.09	0.28	−0.06	0.53
Pre-PAQ/L3 (score)	−0.12	0.06	−0.15	0.08	−0.08	0.35
Pre-PAQ/L4 (score)	−0.02	0.74	−0.06	0.47	−0.01	0.88
Pre-PAQ/L5 (score)	−0.04	0.54	−0.07	0.44	−0.05	0.55
Time spent outdoors (hours/week)	−0.05	0.42	0.01	0.90	−0.15	0.09

*r*—Pearson’s product moment correlation coefficient; *p*—level of significance.

**Table 3:** Correlates of the 25(OH)D level in the total sample (*n* = 260). 25(OH)D sufficiency (>75 nmol/L) was used as the reference value.

	25(OH)D Deficiency (<50 nmol/L)		25(OH)D Insufficiency (50–75 nmol/L)	
Independent Variables	OR	95% CI	OR	95% CI
Gender				
Male	0.33	0.15–0.74	0.60	0.25–1.43
Female	REF		REF	
BMI ^cont^	1.08	0.89–1.31	1.08	0.88–1.31
Pre-PAQ/L1 ^cont^	1.15	0.71–1.86	1.00	0.59–1.70
Pre-PAQ/L2 ^cont^	1.49	0.81–2.75	1.14	0.59–2.20
Pre-PAQ/L1 + L2 ^cont^	1.22	0.86–1.75	1.05	0.72–1.54
Pre-PAQ/L3 ^cont^	1.40	0.76–2.58	0.95	0.49–1.85
Pre-PAQ/L4 ^cont^	1.15	0.68–1.94	0.88	0.49–1.56
Pre-PAQ/L5 ^cont^	2.35	0.84–6.68	1.92	0.65–5.69
Time spent outdoors ^cont^	1.04	0.98–1.10	1.03	0.97–1.10

OR—odds ratio; CI—confidence interval; ^cont^ indicates continuous variables.

**Table 4:** Correlates of the 25(OH)D level in boys (*n* = 132). 25(OH)D sufficiency (>75 nmol/L) was used as the reference value.

	25(OH)D Deficiency (<50 nmol/L)		25(OH)D Insufficiency (50–75 nmol/L)	
Independent Variables	OR	95% CI	OR	95% CI
BMI	1.04	0.82–1.31	1.13	0.88–1.44
Pre-PAQ/L1	1.33	0.71–2.49	0.88	0.44–1.76
Pre-PAQ/L2	1.21	0.59–2.47	1.23	0.58–2.61
Pre-PAQ/L1 + L2	1.22	0.79–1.88	1.02	0.64–1.63
Pre-PAQ/L3	1.45	0.69–3.04	0.85	0.37–1.95
Pre-PAQ/L4	1.05	0.57–1.93	0.83	0.43–1.62
Pre-PAQ/L5	1.96	0.63–6.12	1.88	0.57–6.16
Time spent outdoors	1.01	0.94–1.08	1.02	0.95–1.09

**Table 5:** Correlates of the 25(OH)D level in girls (*n* = 128). 25(OH)D sufficiency (>75 nmol/L) was used as the reference value.

	25(OH)D Deficiency (<50 nmol/L)		25(OH)D Insufficiency (50–75 nmol/L)	
Independent Variables	OR	95% CI	OR	95% CI
BMI	1.13	0.81–1.57	1.01	0.71–1.45
Pre-PAQ/L1	0.99	0.47–2.13	1.1	0.49–2.47
Pre-PAQ/L2	2.21	0.62–7.94	1.07	0.27–4.35
Pre-PAQ/L1 + L2	1.25	0.66–2.36	1.09	0.55–2.17
Pre-PAQ/L3	1.39	0.46–4.17	1.11	0.34–3.68
Pre-PAQ/L4	1.46	0.52–4.08	1.06	0.35–3.26
Pre-PAQ/L5	4.29	0.33–56.45	2.65	0.18–39.51
Time spent outdoors	1.14	0.99–1.30	1.1	0.95–1.27
